# Effectiveness of standardized ultrasound guided percutaneous treatment of lateral epicondylitis with application of autologous blood, dextrose or perforation only on pain: a study protocol for a multi-center, blinded, randomized controlled trial with a 1 year follow up

**DOI:** 10.1186/s12891-019-2711-0

**Published:** 2019-07-31

**Authors:** Renée Keijsers, P. Paul F. M. Kuijer, Koen L. M. Koenraadt, Michel P. J. van den Bekerom, Carina L. E. Gerritsma-Bleeker, Annechien Beumer, Monique H. W. Frings-Dresen, Denise Eygendaal

**Affiliations:** 1 Department of Orthopaedic Surgery, Amphia Hospital, Molengracht 21, P.O. Box 90158, 4800 RK Breda, The Netherlands; 2 Amsterdam University Medical Centers, University of Amsterdam, Coronel Institute of Occupational Health, Amsterdam Public Health Research Institute, Meibergdreef 9, 1105 AZ Amsterdam, The Netherlands; 3 Department of Orthopaedic Surgery, O.L.V.G, Oosterpark 9, P.O. Box 95500, 1090 HM Amsterdam, The Netherlands; 4 Department of Orthopaedic Surgery, Martini Hospital, Van Swietenplein 1, Groningen, 9728 NT The Netherlands; 5 Department of Orthopaedic Surgery, Amsterdam University Medical Centers, Meibergdreef 9, Amsterdam, AZ 1105 The Netherlands

## Abstract

**Background:**

In the treatment of Lateral Epicondylitis (LE) no single intervention concerning injection therapies has been proven to be the most effective with regard to pain reduction. In this trial 3 injection therapies (perforation with application of autologous blood, perforation with application of dextrose and perforation only) will be compared in a standardized and ultrasound guided way. The objective is to assess the effectiveness of these 3 injection therapies on pain, quality of life and functional recovery. By conducting this study, we hope to make a statement on the effectiveness of injection therapy in the treatment of LE. Hereby, unnecessary treatments can be avoided, a more universal method of treatment can be established and the quality of the treatment can be improved.

**Methods/design:**

A multicenter, randomized controlled trial with a superiority design and 12 months follow-up will be conducted in four Dutch hospitals. One hundred sixty five patients will be recruited in the age of 18 to 65 years, with chronic symptomatic lateral epicondylitis lasting longer than 6 weeks, which have concordant pain during physical examination. Patients will be randomized by block randomization to one of the three treatment arms. The treatment will be blinded for patients and outcome assessors. The following three injection therapies are compared: perforation with application of autologous blood, perforation with application of dextrose and perforation only. Injections will be performed ultrasound guided in a standardized and automated way. The primary endpoint is: pain (change in ‘Visual Analogue Scale’). Secondary endpoints are quality of life and functional recovery. These measurements are collected at baseline, 8 weeks, 5 months and 1 year after treatment.

**Discussion:**

When completed, this trial will provide evidence on the effectiveness of injection therapy in the treatment of lateral epicondylitis on pain, quality of life and functional recovery. In current literature proper comparison of the effectiveness of injectables for LE is questionable, due to the lack of standardization of the treatment. This study will overcome bias due to manually performed injection therapy.

**Trial registration:**

This study is registered in the Trial Register (www.trialregister.nl) of the Dutch Cochrane centre. Trial ID; NTR4569. http://www.trialregister.nl/trialreg/admin/rctview.asp?TC=4569

## Background

In the Dutch population seven in 1000 patients visiting a general practitioner (GP) are diagnosed with a lateral epicondylitis (LE) or tennis elbow. The incidence of LE seems independent of sex and ethnical background. Only age influences the incidence with the highest incidence between 40 and 50 years. LE is a disease that is associated with patients in working age from the age of 20 up to 65 [1]. The Dutch Standard on LE for GPs states that about 20% of the patients with LE do not recover within 6 months and 10% not even in 12 months. Each year a GP refers about 2% of the patients with LE to an orthopaedic surgeon [2, 3].

There are several hypotheses regarding the cause of the tendinosis in LE based on histopathological, biochemical and clinical findings. Cell apoptosis, angiofibroblastic features, or abnormal biochemical adaptations, largely suggest that a failed healing response underlies the condition [4, 5]. With respect to the origin more consensus exists, i.e. the Extensor Carpi Radialis Brevis (ECRB) tendon is most commonly affected [6].

Currently, different injectables are used in the treatment of LE without proper scientific evidence [1]. A meta-analysis by Krogh et al. [7] confirmed this statement and found a paucity of evidence from unbiased trials on which to base treatment recommendations for LE. However, this meta-analysis also showed that perforation with application of autologous blood and prolotherapy (injection with dextrose) both seemed more effective than placebo on pain reduction. In addition, these injectables seemed to be more effective after six weeks than corticosteroids on pain reduction.

Autologous blood contains platelets with growth factors that may help in the healing process of chronic injuries. These platelet growth factors stimulate the healing process and lead to partial modification of the damaged tissue. The hypothesis is that these growth factors stimulate angiogenesis and cell proliferation and increase tensile strength and the recruitment of repair cells.

Injection therapy with application of dextrose is a common treatment in chronic musculoskeletal pain, including LE. Animal model studies suggest that the treatment by perforation with application of dextrose may enlarge and strengthen ligament and tendon insertions. The precise mechanism is unclear [8].

Besides the effectiveness of the injectables, the hypothesis is that the needle is used to either break up scars or poke holes in the injured tendon so that bleeding occurs. The blood cells carry precursors, which eventually develop into collagen to replace the damaged tendon. Therefore, this study compares the different injectables with perforation without application of an injectable.

The current debate on studies related to injectables for LE is that in most cases the perforations are performed manually and ‘blindly’ without ultrasound guidance. Moreover, the number and depth of perforations is often not defined. Proper comparison of injectables for LE is therefore questionable [7]. The proposed study compares the effectiveness of perforation of the Extensor Carpi Radialis Brevis (ECRB) tendon to perforation with application of autologous blood or dextrose in the treatment of LE in a standardized and ultra-sound guided way. This overcomes bias due to manually performed injection therapy.

Our hypothesis is that there is no difference in effectiveness between perforation only and perforation with application of one of the injection fluids. The potential health care efficiency gain consists of more homogeneity in the treatment of LE. Hereby, unnecessary treatments can be avoided, a more universal method of treatment can be established and the quality of the treatment can be improved.

The primary objective of this study is to compare the difference in effectiveness of perforation only versus perforation with application of autologous blood or dextrose on pain in the treatment of LE. The primary endpoint is pain on the lateral side of the elbow after a provocation test 5 months after treatment. The secondary objectives are to examine quality of life and functional recovery.

## Methods/design

### Study design

This trial is a multicenter, blinded, three-arm randomized controlled trail with a superiority design with a 12 months follow-up. Data will be presented in line with the CONSORT statement.

### Population

Patients will be recruited in four large teaching hospitals in the Netherlands; Amphia Breda, OLVG Amsterdam, Martini hospital Groningen and Deventer hospital. A total of 165 patients are needed; 55 patients will be included in each of the three trial arms.

#### Inclusion criteria

Patients with chronic symptomatic lateral epicondylitis that have not responded to conservative treatment are eligible to take part when they meet all the following inclusion criteria;

- Symptoms lasting longer than 6 weeks.
- Age from 18 to 65 years.
- Concordant pain during physical examination; pain during palpation of the lateral epicondyle and pain during dorsiflexion of the wrist (from a neutral position and elbow straight) against resistance.
- Unilateral LE (mild cases of LE on the contralateral elbow without functional limitations are allowed).
- Able to read and write in Dutch and should provide informed consent.

#### Exclusion criteria

- Prior injection therapy (during this episode of LE), surgery or trauma at the affected elbow.
- Inflammatory diseases (i.e. rheumatoid arthritis, psoriatic arthritis, or reactive arthritis)
- Other elbow pathology
- Additional pain at the medial epicondyle
- Neck pain or shoulder pain correlated with elbow pain such as C6 radiculopathy or with disability of the arm or other chronic widespread pain syndromes.
- Traumatic onset of LE
- Abnormalities on the X-ray. Abnormal findings are defined as; all conditions that suggest other underlying pathology than LE
- Allergy for lidocaine

The inclusion of the patients will be done by an orthopedic surgeon or trained resident.

### Intervention

**Fig. 1:**
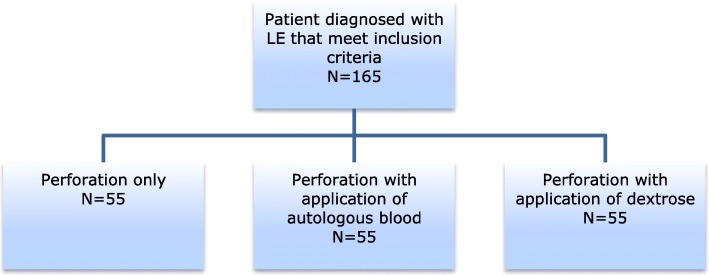
Flowchart study design

The patient will be randomized and included in one of the three treatment arms for lateral epicondylitis (Fig. 1):

- Perforation with application of autologous blood; a venous blood sample is injected in the affected tendon.
- Perforation with application of dextrose: solution with 5 ml 40% dextrose and 3 ml of 0.9% saline and 2 ml of 1% lidocaine.
- Perforation only, without application of a fluid.

**Fig. 2:**
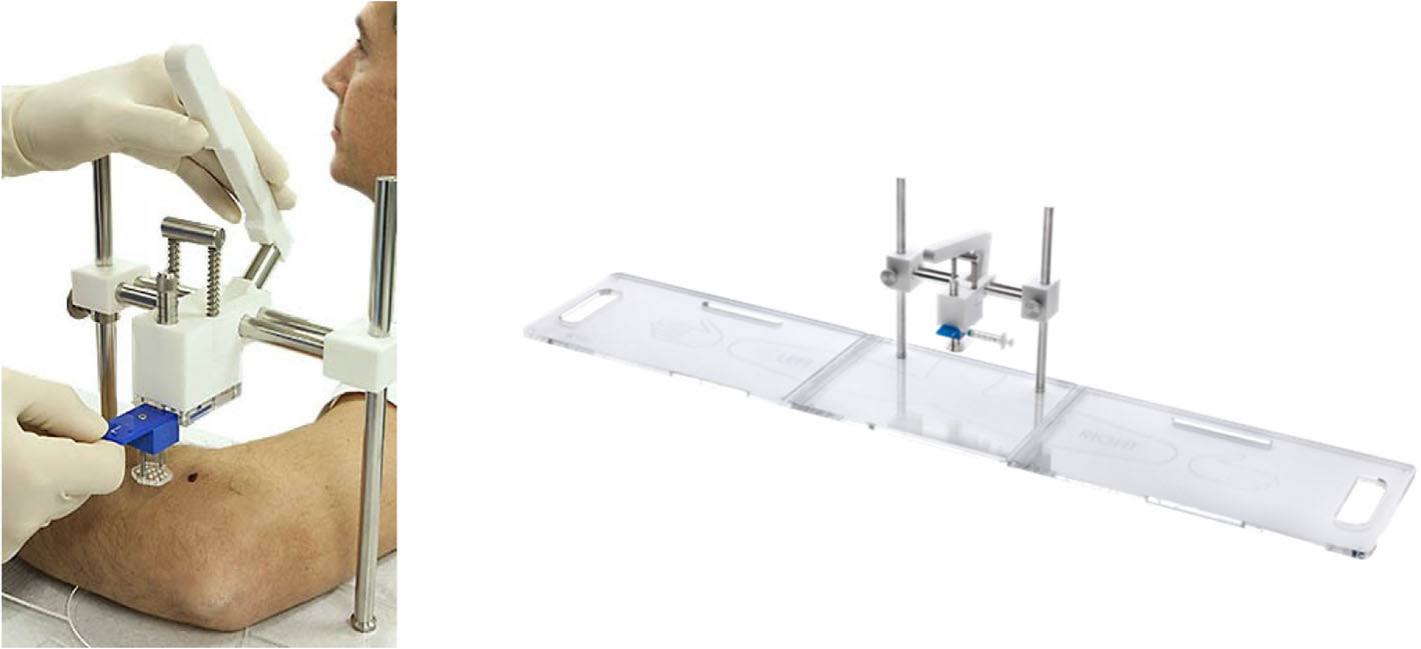
ITEC device (CE 621544, ITEC Medical B.V. Enschede, the Netherlands). Image under copyright by ITEC Medical B.V. and published with their permission

**Fig. 3:**
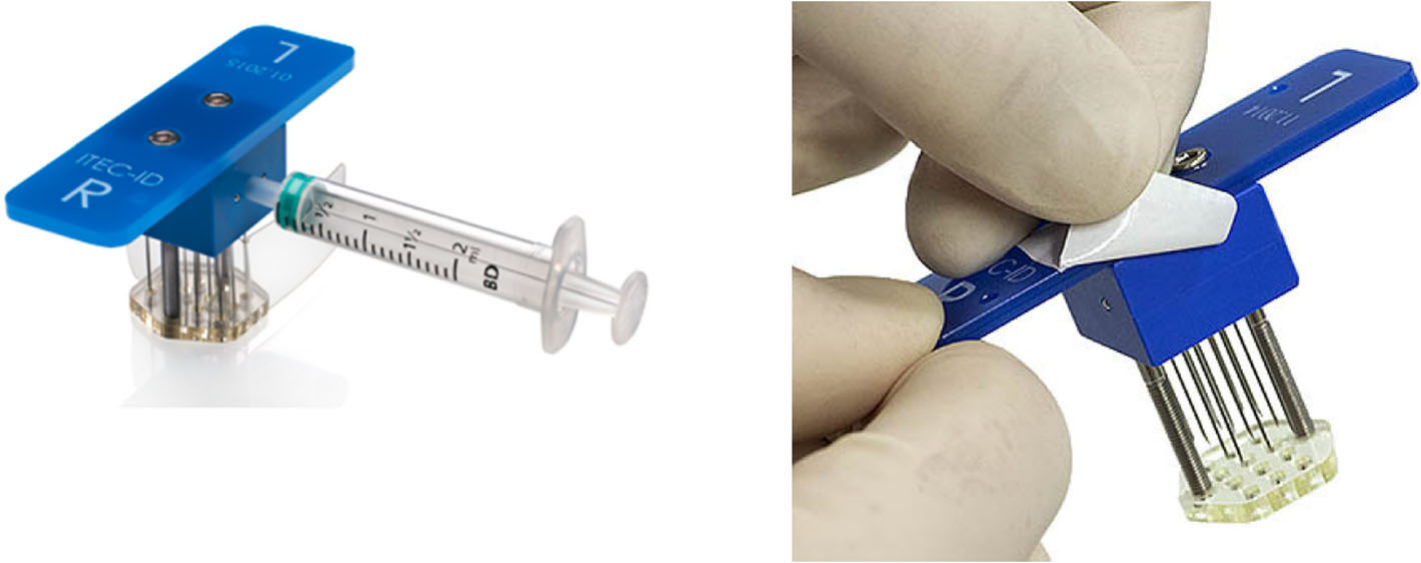
ITEC disposable. Image under copyright by ITEC Medical B.V. (Enschede, the Netherlands) and published with their permission

The percutaneous perforation will be performed by a single perforation at the affected elbow using the ITEC (Instant Tennis Elbow Cure) device (CE 621544, ITEC Medical B.V. Enschede, the Netherlands) (Fig. 2). This device is designed to perforate the Extensor Carpi Radialis Brevis (ECRB) tendon in an automated and standardized way. This device performs percutaneous, reproducible, accurate perforations with application of an exact amount of fluid (Keijsers R, ten Brinke A, de Haan LJ, Bleys RLAW, van den Bekerom MPJ, Eygendaal D. Standardized ECRB perforations, unpublished). This is preceded by an ultrasound-guided localization of the affected ECRB tendon and depth measurement. The device perforates with a set of 12 sterile disposable needles (3 × 4), which are positioned according to anatomic landmarks of the elbow (Fig. 3). A brief instruction is required before using the device (ITEC Medical device user training). All physicians using the device are certificated.

The perforation of the affected tendon is as follows:

- Disinfection of the skin of the elbow
- Positioning of the arm in 90 degrees flexion in the elbow and 90 degrees abduction in the shoulder.
- Depth measurement of the Extensor Carpi Radialis Brevis tendon with an ultrasound probe (5–12 MHz phased array transducer).
- Setting the patient specific depth of the perforation on the device.
- Perforation by a single movement of the arm of the device in which the elbow is infiltrated with one of the injectables.
- Application of 12 drops of the injection fluid (if applicable), with a total amount of 0.4 cc (by a 2 cc syringe).

Before treatment a venous blood sample will be collected from each patient by a venipuncture from the unaffected arm. This sample is used for the treatment of patients in the autologous blood treatment arm and is covered by opaque tape. For the treatment with dextrose or perforation without infiltration this is redundant, but it will be done in order to ensure blinding. The venous blood sample is collected by a supporting nurse or by the physician who performs the treatment.

Co-interventions as visiting a physiotherapist, acupuncturist or osteopath are allowed and will be registered. The use of pain medication besides the needling therapy is allowed and will be registered. After the injection patients are advised to move their arm as they normally do but to avoid lifting and heavy labor for one week.

### Outcome measures and endpoints

#### Demographic variables

Descriptive data will be reported before treatment (baseline) of all participants regarding age, sex, body mass index, dominant arm, job and whether they smoke or not (by the question; do you smoke?). The jobs of the subjects will be classified according to the International Standard Classification of Occupations (ISCO-08).

#### Primary outcome

The main outcome measures are the changes in pain using a Visual Analog Scale (VAS, 0–100) [9]. The VAS consists of a 100-mm horizontal numbered line anchored at one end (0) with the words “no pain” and at the other end (100) with the words “worst pain imaginable.” Patients are asked to score their pain on this line during rest (at time of measure), provocation and maximum grip strength. The provocation test is conducted on the outpatient clinic by resisted dorsiflexion of the wrist during full elbow extension. The VAS pain scores will be measured at baseline and 8 weeks, 5 months and 1 year after treatment.

The main outcome measure is the change in VAS when performing the provocation test 5 months after treatment.

#### Secondary outcomes

Secondary outcome measures are quality of life and functional recovery. Assessments will be made at baseline and 8 weeks, 5 months and 1 year after treatment. For the functional recovery a physical examination is conducted of the affected arm compared to the healthy arm by an orthopaedic surgeon or trained resident. Table 2 shows the items scored at baseline with physical examination. At the 8 weeks and 5 months follow-up visits, a specialized nurse, physiotherapist, trained resident or orthopaedic surgeon (depending on the hospital the patient is treated) will perform a physical examination of both elbows (Table 1). To assess the functional recovery, work ability and patients’ subjective experience on function pain and quality of life the patients are asked to complete a number of questionnaires (Table 2).

**Table 1:** Physical examination

Test	Gradation
At baseline	
Valgus stability	Grade 1/2/3
Pivot shift	Positive/negative
Position of the axis	Normal/valgus/varus
Motor function and sensibility of the ulnar nerve	Intact/disturbed
At baseline, 8 weeks and 5 months	
ROM; Flexion/extension, pronation/supination. Measured by classic goniometry	In degrees
Hydrops	Yes/no
Pain on palpation epicondyle and ECRB;	Yes/no
Pain during dorsiflexion of the wrist (from a neutral position and elbow straight) against resistance	Yes/no
Strength; maximum voluntary hand force measured with a Jamar manual force meter [20]	In Kilogram
Strength; ratio between the affected and healthy arm is calculated. Three consecutive measurements will be performed with one minute intervals between contractions.	In Kilogram The average force of three repetitions will be calculated

**Table 2:** Overview of questionnaire used to assess the functional recovery, work ability and patients subjective experience on function pain and quality of life. The questionnaires will be completed at baseline, 8 weeks, 5 months and 1 year after treatment

Measures	Questionnaires	Scores	Reliability and validity	Population
Physical function and symptoms	Quick-DASH [10–12]	0 (no disability) to 100 (most severe disability)	Cronbach α > or = 0.92 and an ICC^a^ > or = 0.94. validity was established (r > or = 0.64	Patients with various upper-limb conditions
Patients’ subjective experience of elbow surgery on elbow function, pain, and Quality of life	Oxford Elbow score [13]	0 (unsatisfactory joint function)to 48 (satisfactory joint function)	Cronbach’s α coefficients for the function, pain and social-psychological domains respectively 0.90, 0.87 and 0.90,. ICC 0.87 for function, 0.89 for pain and 0.87 for social-psychological.	Patients after elbow trauma and surgery
Functional recovery	The WORQ-UP questionnaire for the upper limb [14]	0 (no disability) to 102 (most severe disability)	-	Patients with elbow complaints
Pain and functional ability	The Dutch Patient-Rated Tennis Elbow Evaluation PRTEE-D [15]	Pain subscale (0 = no pain, 10 = worst imaginable). function subscale (0 = no difficulty, 10 = unable to do)	Crohnbach’s α 0.98 and 0.93 for the pain subscale and 0.97 for the function subscale. ICC 0.98 and 0.97 for pain and function scale	Patients with LE
Work ability	The first question on work ability of the Work Ability Index amended for elbow pain “current work ability compared with the lifetime best regarding your elbow complaints”, [16–18]	0 (completely unable to work) to 10 (work ability at its lifetime best)	Acceptable test-retest reliability and predictive validity for return to work	Construction workers with and without musculoskeletal complaints of the upper extremity, lower back, and lower extremity
Generic measure of health gain, as a derivative of quality of life	Quality adjusted life years (EQ-5D/QALYs) [19]	a year of life lived in perfect health is worth 1 QALY and a year of life lived in a state of less than this perfect health is worth less than 1.	validated, no studies on reliability	General population sample

^a^*ICC* The intra-class correlation coefficient

The 1-year follow-up moment will be conducted by email or, if necessary, by telephone by a trained investigator. The patient will be asked to complete the questionnaires mentioned above. All complications and/or co-interventions will be registered.

#### Crossover

Patients treated with perforation without infiltration which could not be classified as “success” should also be treated with infiltration with autologous blood, a so called one arm crossover. This is currently the most common treatment of LE in OLVG and Amphia hospital. A treatment is classified as a success when there is a reduction of pain of 10 points (0-100 mm VAS score) 5 months after the treatment compared with baseline. If the treatment wasn’t successful after 5 months the given treatment can be looked up in an encrypted list; this list only shows if the patient has had an infiltration or perforation, without the specification of the injectable. After the crossover moment, patient data will be collected as described above.

### Sample size and power calculation

A reduction in pain of at least 10 mm on a 0–100 mm visual analogue scale is deemed a clinical significant improvement. Due to the three study arms of our RCT, there are also three null hypotheses tested:| A - B | ≥ 10 | A - C | ≥ 10 and | B - C | ≥ 10 points on the VAS pain scale, according to a superiority design. In which A, B and C stands for dextrose, autologous blood and perforation only respectively. A number of 47 patients is required in each trial arm when a two-sided 98% confidence interval is used, with a two-sided alpha of 2%, corresponding to a z-value of 2.34 (J Scott Armstrong: Tables for Statistical Significance with Multiple Comparions). To adjust for a maximum of 10% dropout over the one year follow up period, 55 patients will be included in each of the three trial arms. Therefore, a total of 165 patients are needed. It is expected that each week in both hospitals a total of 3 patients can be included. Therefore, the inclusion period takes about 55 weeks. Patients that are withdrawn from the study will not be replaced, since there is accounted for these dropouts in the power analysis.

### Recruitment and enrollment

All consecutive patients presenting to the department of orthopedic surgery with LE who meet the inclusion criteria will be invited to participate in the trial. The treating surgeon or resident will introduce the trial to the patient and address the patient’s questions. Information will be handed to the patient to read at home. If the patient is willing to participate, informed consent will be obtained. Participants may take as long as they like to consider participation, provided that they still meet all eligibility criteria.

#### Randomization

After providing informed consent, eligible patients will be randomized by block randomization to one of the treatment arms, in order to have similar groups at any time.

Pain reduction seems dependent on the patient-reported activity or work-relatedness of this complaint [21, 22]. Therefore, patients with work related complaints have to be equally present in the intervention and in the care-as-usual groups. This is secured by block randomization using the following question:

#### My complaints of the elbow are caused or aggravated by my work (or household activities)

The answering categories are; totally agree, agree, agree nor disagree, disagree, totally disagree.

Patients are grouped in two groups;

- Totally agree, agree
- agree nor disagree, totally disagree and disagree.

Two randomization lists will be conducted by computer by means of a pseudo random number generator, stratified into two strata to overcome differences in groups due to physical job demands.

The preparation of the randomization envelopes will be done by a research coordinator of the Amphia hospital, who is no member of the research team and the closed envelops will be distributed to the contributing hospitals. The envelopes will be opened in order of their consecutive numbers just before treatment.

#### Blinding

The treatment will be blinded for both the patient and outcome assessor (the specialized nurse, physiotherapist or trained resident). During the injection the patient will be blinded. Injectables will be blinded by a supporting nurse through the use of an opaque tape. The outcome assessors are different for each participating center. However, the outcome assessors are always blinded during all the follow up visits. The physician who performs the perforation does not have to be blinded, as long as he or she does not perform the follow-up visits.

#### Missing data

The primary outcome measure ‘pain’ will be plotted against the different follow up moments to determine whether the complete cases differ from the dropouts of the three groups. Potential differences are tested. Depending on whether there are significant differences associated with the drop-outs, a mixed model analysis with or without covariates will be used. For a mixed model analysis, no imputation of missings is required.

### Statistical analysis

To compare groups the three groups (A – B, A – C and B - C) Chi-square tests will be used for categorical variables, and independent t-tests for continuous variables. The primary aim is to assess whether the reduction in pain for perforation with application of autologous blood or dextrose is more than 10 points compared to perforation only.

If so, a comparison will be made between the reduction in pain for perforation with application of autologous blood versus dextrose. A mean and a two-sided 98% confidence interval will be calculated. A mixed model will be used taking into account effects of time and treatment. The analyses are primarily performed according to the intention-to-treat principle.

In detail, data will be analyzed in the per protocol (pp) set as well as in the intention-to-treat (itt) set of patients. The pp-set consists of all patients, categorized according to the treatment eventually received, with non-missing observations on the pain VAS at all three visits during follow-up (8 weeks, 5 months and 1 year). The itt-set consists of all randomized patients.

To get a better understanding, a linear mixed model analysis will be performed with the three repeated measurements of pain VAS as dependent variables. Along with treatment (a three-level nominal variable) the following covariables will be included in the model as independent variables: baseline pain VAS, the binary variable perceived aggravation of pain due to work ((totally) agree vs otherwise) and a three-level nominal variable time (visit number). Moreover, the interaction between treatment and time will be tested. If this interaction is not significant at the 0.01 level, then one overall treatment effect will be estimated assuming parallelism of the treatment effect. If this interaction turned out to be significant at the 0.01 level, then the effect of the 5 months’ visit will be considered the main effect of interest. Effects will be quantified by estimating two-sided 98% confidence intervals of the pair wise mean differences in pain VAS between the three treatment groups. Equivalence between two treatments is accepted if such a confidence interval is totally overlapped by the interval − 10 + 10. A restricted maximum likelihood estimation method will be used to estimate the coefficients so that missing values are properly imputed in order to meet the itt-condition. Under the pp-condition only patients with non-missing observations will enter the linear mixed model.

Similar analysis will be performed for functional recovery and quality of life. Otherwise the descriptives of the secondary outcome measures will be reported for the three groups.

### Adverse events

Adverse events (AEs) are defined as any undesirable experience occurring to a subject during the study, whether or not considered related to the needle therapy. All adverse events reported spontaneously by the subject or observed by the investigator or his staff will be recorded. The expected side effects are similar to a normal injection, such as temporarily worsening of the symptoms and any additional pain caused by the injection therapy itself. However, these side effects are not specific for this method. The ITEC device mimics the normal treatment of lateral epicondylitis with manual injection therapy performed by an orthopaedic surgeon. A pilot study, performed at the Amphia hospital Breda, the Netherlands, concerning 25 patients treated with perforation therapy with the ITEC device reported no adverse device effects.

All AEs will be monitored until they have abated, or until a stable situation has been reached in which no changes are expected. Depending on the event, follow up may require additional tests or medical procedures as indicated, and/or referral to the general physician or a medical specialist.

Subjects can leave the study at any time for any reason if they wish to do so without any consequences. The investigator can decide to withdraw a subject from the study for urgent medical reasons.

## Discussion

In current literature, comparisons of the effectiveness of injectables for LE are questionable due to the lack of standardization of the treatment; most injections are performed manually and ‘blindly’ without ultrasound guidance and the amount of injected fluid and number and depth of perforations is often not defined. This is aggravated by the lack of understanding the pathophysiology of LE and therefore the mechanism of action of these injectables on the tendon and on the patients’ symptoms. [23] This study is therefore set up in a standardized and ultrasound guided way. This overcomes bias due to manually performed injection therapy.

This study compares three different treatment options for LE; perforation of the Extensor Carpi Radialis Brevis (ECRB) tendon only, perforation with application of autologous blood and perforation with application of dextrose. Besides the injectables used in this study, several novel injection therapies are used in recent research such as platelet rich plasma (PRP) and the sclerosing agent polidocanol. [24, 25] These might have a potential benefit in the treatment of LE. Platelet-rich plasma is prepared from autologous whole blood, which is centrifuged to concentrate platelets in plasma. Polidocanol is used to sclerose areas of high intratendinous blood flow. This neovascularity might be associated with the underlying pathophysiology of LE. [26, 27] In the study of Branson there were no differences in effectiveness between autologous blood and polidocanol. [24] Currently there is insufficient evidence that treatment of LE with PRP is more effective than autologous blood. [7, 28] Besides these novel injectables, steroids are still commonly used in the treatment of LE. This is remarkable because its effects on the long term are worse than other injection therapies or a wait- and- see policy. [24, 29]

There is a paucity of evidence on the effectiveness of all injectables used in the treatment of LE and it is still questionable if there is a major benefit of injection therapies given the self-limiting nature of the condition. [7] Therefore one of the treatment arms in this study does not include an infiltration but is perforation only. It is hypothesized that bleeding occurs in the affected ECRB tendon by poking holes with the needles. This blood cells carry precursors, which eventually could develop into collagen to replace the damaged tendon. No placebo group is included in this study. This has been chosen because of the expected inclusion difficulties. In our experience it is challenging to motivate a patient to participate in a study with a placebo group. Especially when the complaints are disabling and persistent.

For practical reasons, it was chosen not to include all different injectables. Due to the self-limiting character in the majority of cases, the number of participants needed to demonstrate a clinically relevant difference would be too high for feasibility. Ideally, in future studies all different injection therapies should be compared to a placebo or sham injection group. However, we think our study takes the most important injection therapies into account and given the standardized design, this study will be a valuable addition to current literature.

## Data Availability

The datasets used and/or analyzed during the current study are available from the corresponding author on reasonable request.

## Abbreviations

AEs: Adverse events
ECRB: Extensor Carpi Radialis Brevis
GP: General practitioner
ITEC: Instant Tennis Elbow Cure
ITT: Intention-to-treat
LE: Lateral epicondylitis
PP: Per protocol
PRP: Platelet rich plasma
VAS: Visual Analog Scale
